# Carcass-Based Surveillance of Amphibian Herpesviruses, Ranaviruses and *Batrachochytrium dendrobatidis* in Schleswig-Holstein, Northern Germany

**DOI:** 10.3390/pathogens15030286

**Published:** 2026-03-06

**Authors:** Natalie Steiner, Lotte C. Striewe, Christoph Leineweber, Sara Grau Camps, Peter Wohlsein, Frederik Elze, Simon Rohner, Rachel E. Marschang, Ursula Siebert

**Affiliations:** 1 Institute for Terrestrial and Aquatic Wildlife Research (ITAW), University of Veterinary Medicine Hannover, 25761 Buesum, Germany; 2 Laboklin GmbH & Co., KG, 97688 Bad Kissingen, Germany; 3 Department of Pathology, University of Veterinary Medicine Hannover, 30559 Hannover, Germany; 4 Aquatic Ecology, Faculty of Biology, University of Duisburg-Essen, 45141 Essen, Germany

**Keywords:** amphibian disease surveillance, post-mortem diagnostics, amphibian herpesviruses, chytridiomycosis, ranaviruses, common toad, *Bufo bufo*, common frog, *Rana temporaria*

## Abstract

Amphibian populations are undergoing dramatic global declines, with infectious diseases recognized as major contributors. While chytridiomycosis, caused by *Batrachochytrium dendrobatidis* (*Bd*), and disease caused by ranaviruses are well known, herpesviruses in amphibians remain comparatively neglected. We conducted a passive survey using carcasses collected between 2022 and 2025 in Schleswig-Holstein, northern Germany. Dead amphibians (*n* = 187) were dissected. Skin, kidney, liver, and brain samples were screened for *Bd*, ranaviruses, bufonid herpesvirus 1 (BfHV1), and ranid herpesvirus 3 (RaHV3) by PCR. BfHV1 was detected in nearly half of the *Bufo bufo* (common toads; 48.6%), while RaHV3 was identified in 23.6% of the *Rana temporaria* (common frogs) examined. *Bd* was detected in a single *B. bufo*, while ranaviruses were not detected. BfHV1 was present in skin, liver, kidney, and brain samples, whereas RaHV3 was detected exclusively in skin samples. No macroscopical or histological lesions characteristic of herpesviruses or *Bd* were found. However, the carcass-based approach frequently limited detailed examination due to compromised sample quality. Our findings confirm the presence of amphibian herpesviruses (BfHV1 and RaHV3) in the region without associated lesions, raising questions about their potential to cause systemic infections. Furthermore, our results highlight the limitations of carcass-based surveillance and underscore the need for complementary diagnostic approaches, such as immunohistochemistry and meta-omics.

## 1. Introduction

Amphibians are currently the most threatened vertebrate group worldwide, with extinction rates estimated to be up to 200 times higher than background levels [1,2]. Declines have been ongoing since the mid-20th century and are driven by various factors, including habitat loss, climate change, introduction of invasive species, and overexploitation [2,3]. Infectious diseases are among the most widely reported causes of the rapid decline of amphibian populations [4].

Declines are also evident in Germany, where several studies have documented the occurrence of major amphibian pathogens. The chytrid fungus *Batrachochytrium dendrobatidis* (*Bd*) is widely distributed throughout the country, with local prevalences of 3–8% [5,6]. In contrast, *Batrachochytrium salamandrivorans* (*Bsal*) has emerged as a major driver of salamander declines, particularly in southern and western Germany [7,8], with increasing evidence of northward spread into adjacent regions [9]. Ranaviruses, by comparison, have only been sporadically detected in Germany [10].

Experimental and field studies in Germany and adjacent European countries indicate substantial variation in host susceptibility, infection dynamics, and disease outcomes for these pathogens [6,11]. The two chytrid fungi differ in host preference, with *Bd* predominantly infecting anuran species and *Bsal* primarily affecting urodeles [12]. Mortality rates vary widely between species and populations and are influenced by environmental conditions, host immune responses, and pathogen load. Routes of infection include direct contact between hosts, exposure to contaminated water bodies, and anthropogenic translocation of infected animals or equipment [13].

In natural populations, amphibians are frequently exposed to more than one pathogen simultaneously [14]. Co-infections involving chytrid fungi, ranaviruses, bacteria, and helminths have been increasingly reported and are thought to exacerbate disease severity and mortality through synergistic effects on host immunity and tissue damage [15,16]. In particular, interactions between fungal and viral pathogens may increase host susceptibility and facilitate systemic infections, highlighting the importance of investigating pathogen assemblages rather than single agents in isolation [17].

In addition to the chytrid fungi and ranaviruses, several herpesviruses belonging to the family *Alloherpesviridae* have been described in amphibians, including ranid and bufonid herpesviruses [18,19,20,21,22]. These viruses exhibit considerable variation in host range and disease expression, ranging from subclinical infections to proliferative, ulcerative, and systemic disease [23].

In Germany, BfHV1 and RaHV3 are the most frequently reported and clinically relevant amphibian herpesviruses, with confirmed infections in free-ranging bufonid and ranid species [24], including in Schleswig-Holstein, where PCR detection rates in live-animal sampling reach up to 60% for BfHV1 in *Bufo bufo* Linnaeus, 1758 (common toads) and 44% in *Rana temporaria* Linnaeus, 1758 (common frogs) [25]. Other herpesvirus lineages, such as RaHV1 and RaHV2, have either not been reported from Europe or appear to be restricted to specific host species and geographic regions, and are therefore considered of limited relevance for native amphibian populations in Germany [22].

Despite the growing body of research on amphibian pathogens in Germany and neighboring regions, systematic data on their occurrence, co-occurrence, and population-level impact remain limited, particularly in northern Germany. Schleswig-Holstein features rich amphibian diversity and is currently known to be part of the native range of 15 amphibian species [26], yet systematic health monitoring is lacking. Carcass-based, non-invasive sampling provides a valuable approach in such contexts, as it allows for pathogen detection without further impacting vulnerable populations. Moreover, sampling deceased animals can capture advanced clinical or pathological signs that are often missed in live-animal surveys [27]. Additionally, carcass-based surveillance preferentially samples deceased animals and therefore more frequently captures advanced biological or clinical changes than live-animal sampling [28].

The aim of the present study was to address these knowledge gaps by opportunistically testing dead anurans collected across Schleswig-Holstein for *Bd*, ranaviruses, BfHV1, and RaHV3. *Bsal* was excluded because it primarily affects urodeles. A further objective was to evaluate the utility of carcass-based surveillance as a complementary tool for amphibian health monitoring, offering a non-invasive and cost-effective method to detect infections in wild populations.

## 2. Materials and Methods

### 2.1. Animal Collection

**Figure 1:**
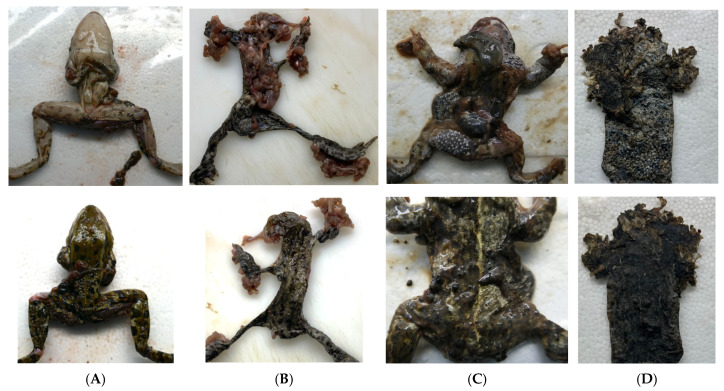
Necropsy images of collected amphibians. Ventral (**upper row**) and dorsal (**lower row**) views are shown for each individual. Animal (**A**) was found near water and was not believed to be roadkill, whereas animals (**B**–**D**) were believed to be roadkill. Species identification was as follows: (**A**)—*Pelophylax* spp. (water frog), (**B**)—*Bufo bufo* (common toads), and (**C**,**D**)—*Epidalea calamita* (natterjack toad). Animals exhibited varying degrees of decomposition: (**A**,**B**) were classified as decomposition state 3 (active decay), (**C**) as state 4 (advanced decay), and (**D**) as state 5 (dry/skeletonized). Common necropsy findings included detachment of the upper skin layer (**A**–**C**), severe protrusion and damage of internal organs (**B**,**C**), and contamination of internal organs with soil or organic material (**C**). Individuals in decomposition state 5 (**D**) were excluded from further analyses.

A total of 187 dead anurans, representing nine species, were collected opportunistically between April 2022 and June 2025 from various locations across Schleswig-Holstein, Germany, as part of research projects aiming to establish a health monitoring program of amphibian populations (see Results 3.1 for species composition and relative abundance). Specimens were provided by volunteers, who recorded metadata including collection coordinates, date, and environmental context (e.g., proximity to roads or water bodies) following a standardised questionnaire. Carcasses were either processed immediately or stored frozen at −20 °C for up to 400 days (mean storage time: 72 days) prior to necropsy. Necropsies were performed on all animals according to a standard protocol. Each animal was identified by species, sex, and life stage, if possible. Decomposition status was assessed based on the criteria described by Brooks [29], summarized as follows: decomposition state 1 (fresh), characterized by the absence of discoloration or insect activity. State 2 (early decay), showing minor grey to green discoloration and skin slippage; state 3 (active decay), marked by discoloration of multiple organs, state 4 (advanced decay), with moist tissue decomposition and sagging of flesh; and state 5 (dry/skeletonization) presenting with mumification and/or skeletonization (examples see Figure 1). Animals with a decomposition status of 5 were excluded from the study. Samples from skin, liver, kidney, and brain were collected, if available, for pathogen screening and histological examination. Brain samples were collected from only a subset of animals at the end of the sampling period following a modification of the sampling protocol during the study.

### 2.2. Molecular Testing

Skin, liver, kidney, and brain samples were screened for *Bd*, BfHV1, RaHV3, and ranaviruses by PCR. Analyses were conducted at Laboklin GmbH & Co. KG (Bad Kissingen, Germany). Samples were stored at −20 °C to −70 °C and shipped on cold packs in insulated boxes. DNA was extracted using the MagNA Pure 96 System with the DNA and Viral NA Small Volume Kit (Roche, Penzberg, Bavaria, Germany) following the manufacturer’s instructions, including internal extraction controls (DNA or RNA Process Control Detection Kit, Roche Diagnostics GmbH, Mannheim, Baden-Württemberg, Germany) to confirm successful nucleic acid isolation and exclude PCR inhibition.

*Bd* detection was performed by real-time PCR targeting the ITS region as described by Boyle et al. [30], and samples were additionally analysed using a lineage-specific PCR targeting the Global Pandemic Lineage (GPL) according to Garland et al. [31]. BfHV1 and RaHV3 were detected using TaqMan real-time PCR assays adapted from Origgi [19] (p. 2) and Origgi [32] (pp. 1228–1229), with primers and probes optimized to an annealing temperature of 60 °C (TIB Molbiol Syntheselabor GmbH, Berlin, Germany) [13].

Ranaviruses were analysed by real-time PCR targeting the major capsid protein gene as described previously [33]. Positive controls for *Bd*, BfHV1 and RaHV3 consisted of clinical samples confirmed by sequencing. The *Bd*-positive control was obtained from an *Ambystoma mexicanum* (axolotl), the BfHV1-positive control from *B. bufo*, and the RaHV3-positive control from *R. temporaria*. The ranavirus-positive control was a ranavirus isolate propagated in cell culture. Concentrations of all positive controls were adjusted to assay-specific threshold cycle (Ct) values of 30–32 using PCR-grade water. Negative controls consisted of PCR-grade water. Positive and negative controls were included on each PCR plate.

All real-time PCR assays were performed using commercial TaqMan master mixes according to the manufacturers’ recommendations and under the cycling conditions specified in the original publications. Samples were considered positive when amplification occurred within the Ct range defined by the validated diagnostic protocols of the testing laboratory. Ct values < 35 were considered as positive, 35–38 as low positive and >38 as negative for all PCR analyses including positive controls. The analytical sensitivity and limits of detection of all assays have been reported previously in the respective original publications [19,30,31,32,33].

### 2.3. Histology

Where possible, representative samples of ventral and dorsal skin, liver, and kidney were fixed in 4% neutral buffered formalin for at least 24 h, processed routinely, embedded in paraffin, and sectioned at 2 µm. Sections were stained with hematoxylin and eosin and examined by light microscopy (Olympus BX53, Olympus Europa SE & Co. KG, Hamburg, Germany). Brain samples were not collected for histological examination due to extensive liquefaction.

Samples were included in the histological evaluation only if they were preserved in a condition that allowed detailed assessment of the skin layers. Samples showing signs of advanced decomposition were excluded. Criteria for exclusion included loss of epidermal integrity, cytoplasmic and nuclear fading, loss of cellular detail, and pronounced vacuolization, as well as extensive areas of epithelial cells exhibiting karyolysis, karyorrhexis and cytoplasmic swelling [34].

Skin samples were assessed based on the criteria described for an infection with BfHV1 or RaHV3 including the presence of intranuclear inclusion bodies, epidermal hyperplasia, intercellular edema (spongiosis), intracellular and intranuclear vacuolization along with parakeratosis and dyskeratosis [19,23]. Lesions consistent with *Bd* infection such as epidermal hyperplasia, hyperkeratosis and the presence of zoosporangia were also assessed [15].

### 2.4. Statistical Analysis

Statistical analyses were conducted in R (R (version 2024.12.1+563, R Foundation for Statistical Computing, Vienna, Austria)). Associations between pathogen detection (BfHV1, RaHV3, *Bd*) and host or sampling factors—including species, month of collection, storage duration, carcass origin (roadkill vs. other), decomposition state and sex—were assessed using contingency table analyses. For analyses restricted to *B. bufo*, species was not included as a factor. For 2 × 2 tables, Fisher’s exact tests were applied; for larger tables, Chi-square tests were used, with Fisher’s exact test as a fallback in cases of low expected counts. For storage duration, the Wilcoxon rank-sum test was used to compare the continuous numeric storage duration with the binary pathogen presence (negative/positive). Raw *p*-values were adjusted for multiple comparisons using the Benjamini–Hochberg method, and these adjusted *p*-values are reported as FDR. Adjusted *p*-values ≤ 0.05 were considered significant.

Conditional proportions were calculated to assess tissue-specific pathogen prevalence, representing the percentage of positive results within each tissue among individuals testing positive in at least one organ.

Spatial distribution of the collected animals was visualized using R packages sf, ggplot2, and rnaturalearth.

## 3. Results

### 3.1. Species Composition

Species collected during this study included *B. bufo* (*n* = 148), *R. temporaria* (*n* = 19), *Pelophylax* spp. (water frogs, *n* = 8), *Rana arvalis* Nilsson, 1842 (moor frog, *n* = 5), *Epidalea calamita* Laurenti, 1768 (natterjack toad, *n* = 3), *Pelobates fuscus* Laurenti, 1768 (common spadefoot toad, *n* = 2), and *Hyla arborea* Linnaeus, 1758 (European tree frog, *n* = 2).

### 3.2. Pathogen Testing

Only two species—*B. bufo* and *R. temporaria*—tested positive for at least one pathogen.

BfHV1 was detected exclusively in *B. bufo* (Table 1). Most positive results were obtained from skin samples, whereas detection in kidney, liver, and brain occurred less frequently (Table 2). In nearly all positive individuals, the virus was detected in the skin, and only a single animal showed positivity in kidney tissue alone.

Among BfHV1-positive *B. bufo*, only a subset had all four tissue types available. In these individuals, BfHV1 was most often detected in a single tissue, followed by detection in two tissues, whereas fewer animals showed positivity in more than two tissues.

Because brain samples were only available for a subset of animals, excluding brain tissue increased the number of individuals with complete tissue sets. When only skin, kidney, and liver were considered, a similar pattern was observed, with most animals showing positivity in a single tissue type, followed by multi-tissue detection.

RaHV3 was detected in *R. temporaria* and in a single *B. bufo* (Table 1). In all positive individuals, viral DNA was detected exclusively in skin tissue.

*Bd* was detected only in the skin of a single *B. bufo*. Lineage-specific testing identified the *Bd* GPL lineage. Ranaviruses were not detected in any sample.

### 3.3. Decomposition, Sex, and Roadkill

Most of the specimens were rated as decomposition status 3 (35.6%) or 4 (32.4%). Decomposition status of 1 was seen in 0.5% (1/188) and a status of 2 was seen in 28.7% (54/188). Sex could be determined in 146 animals, with 86 males (45.7%) and 60 females (31.9%); while sex was undetermined in 42 individuals (31.6%). Roadkill was suspected in 143 cases (70.1%); 40 (66.7%) females and 71 (82.6%) males. Roadkill was especially high in *B. bufo* with 89.9% (133/148) of collected individuals.

### 3.4. Correlations with Pathogen Detection

To account for uneven sampling among species, statistical analyses were first conducted on the full dataset including all amphibian species and then restricted to *B. bufo* alone. In the full dataset, pathogen detection (BfHV1 and RaHV3) was significantly associated with roadkill status (*p* < 0.001), species (*p* < 0.001), and sampling month (*p* ≤ 0.05), with prevalence peaking in March compared to April and May. However, only *B. bufo* tested positive for BfHV1 and *Bd*; all other species were negative. Additionally, sample sizes for non-toad species were very small. When analyses were restricted to *B. bufo*, the positive association between roadkill and pathogen detection was no longer significant, while the effect of sampling month remained, with higher infection rates in March compared to April and May.

After multiple-testing correction, no significant associations with decomposition state, storage duration or sex were observed. Results of statistical analyses on *B. bufo* are presented in Table 3.

**Figure 2:**
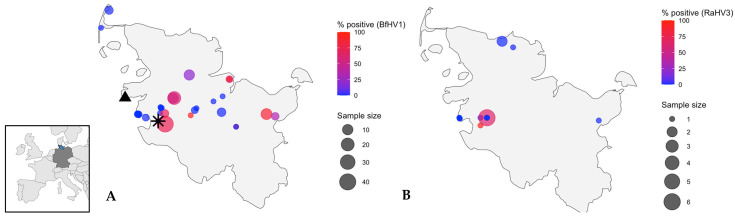
Geographic distribution of amphibians collected in Schleswig-Holstein, northern Germany. The inset map shows the location of Schleswig-Holstein within Europe. (**A**) *Bufo bufo* (common toads) and (**B**) *Rana temporaria* (common frogs). Circle size represents the number of animals sampled at each location; circle colour shows the percentage of individuals testing positive for bufonid herpesvirus 1 (BfHV1) in toads and ranid herpesvirus 3 (RaHV3) in frogs. The asterisk marks the *B. bufo* tested positive for RaHV3; the triangle indicates the *B. bufo* that tested positive for *Batrachochytrium dendrobatidis* (*Bd*).

The location of sampling was significantly associated with BfHV1 status across all amphibians (*p* < 0.001) and within *B. bufo* (*p* < 0.001). However, sampling effort was uneven across sites, with some sites contributing many carcasses and others only a few. Several sites yielded only single individuals. An overview of the distribution of collected *B. bufo* and *R. temporaria* and infection status is provided in Figure 2.

### 3.5. Histology

Histological assessment of the skin was markedly limited by sample degradation and frequent artefacts, including sectioning artefacts and partial loss of the superficial epithelial layers. After exclusion of poor-quality material, only a subset of samples remained assessable (ventral skin: 62/188, 33%; dorsal skin: 100/188, 53%). Occasional focal epithelial hyperplasia was observed; however, these changes could not be reliably distinguished from artefacts. No lesions indicative of any of the pathogens tested were identified, and no association was detected between PCR positivity and any histological changes.

## 4. Discussion

This study demonstrates a high detection rate of herpesvirus infections among amphibians in Schleswig-Holstein, with BfHV1 detected in nearly half of the *B. bufo* and RaHV3 identified in more than a quarter of the *R. temporaria* examined. In contrast, *Bd* was found at a much lower rate, and ranaviruses were not detected at all. Given the carcass-based, opportunistic nature of the sampling, these values should be interpreted as pathogen detection rates in submitted specimens rather than as estimates of true population prevalence.

The high rate of BfHV1 detection in *B. bufo* is consistent with previous reports of 30–60% prevalence in live animals in Germany [24,25,35], confirming that BfHV1 is widely distributed across the country. The higher detection rate in March coincides with the spawning season and may reflect seasonally driven host behaviour and immune modulation, supporting earlier findings of seasonal dynamics in amphibian herpesvirus infections [19,35].

Despite the high rate of PCR-positivity, no gross or histopathological lesions typical of herpesvirus infection, such as intranuclear inclusion bodies [23], were observed. This finding must be interpreted with caution as most of the animals sampled were roadkill. Tearing of the skin and loss of the upper layer of the epidermis were found in most animals, preventing a detailed histological assessment of the skin.

Overall, the histological data obtained in this study did not allow the identification of BfHV1-, RaHV3-, or *Bd*-associated skin lesions in any of the PCR-positive animals.

Environmental conditions, particularly temperature, are known to have a strong influence on herpesvirus replication dynamics. Studies on fish herpesviruses have demonstrated that viral replication, latency, and disease manifestation are temperature-dependent, with reactivation often occurring under thermal conditions optimal for viral replication [36,37,38]. These effects are thought to result from temperature-driven changes in the physiology and immune competence of poikilothermic hosts such as fish and amphibians [39].

In the present study, no environmental data were collected, and site-specific conditions such as temperature, humidity, or habitat characteristics are therefore not available. These limitations are inherent to opportunistic, citizen-submitted sampling and preclude assessment of how environmental factors may influence pathogen detection or infection dynamics. Future investigations should integrate environmental parameters, including vegetation, water quality, and climatic data, to empirically assess their role in viral activation and transmission in wild amphibian populations.

In addition to skin samples, BfHV1 DNA was detected in liver, kidney, and brain tissues. Systemic infection has previously been reported for BfHV1, with viral particles observed in the brain and other internal organs [19]. Severe autolysis of internal tissues in the animals examined in our study prevented histological confirmation of inclusion bodies or other indicators of active infection. The finding of viral DNA in various tissues should therefore be interpreted cautiously. Many of the individuals with BfHV1-positive internal organs were roadkill. They exhibited extensive trauma and organ protrusion, which may have led to contamination of internal tissues with skin material or environmental debris (Figure 1). Future carcass-based studies should prioritize well-preserved specimens with intact body cavities. This would minimize contamination risks and allow more accurate histopathological assessment and help evaluate the possibility of multi-organ involvement in BfHV1 infections.

RaHV3 was mainly detected in *R. temporaria*, with a single positive *B. bufo*. The detection rate in our samples was slightly lower than previously reported from live sampling at four locations in Schleswig-Holstein with 43.86% RaHV3-positive *R. temporaria* [15]. Viral DNA was found exclusively in skin samples, consistent with current knowledge that RaHV3 primarily infects epidermal tissues [9]. In contrast to the *B. bufo* in which BfHV1 was detected in various tissues, none of the *R. temporaria* in which RaHV3 was detected were roadkill, and all had been found dead with intact bodies. Contamination of internal tissues was therefore much less likely in these animals than in the BFHV1-positive *B. bufo*.

Although detections of batraviruses in non-typical host species have occasionally been reported [15], the present dataset does not allow conclusions regarding host specificity or cross-species transmission. Our findings merely confirm that both BfHV1 and RaHV3 occur in sympatric amphibian species, while the true host range remains to be elucidated through targeted experimental or molecular studies. Assertions of strict host specificity among amphibian alloherpesviruses are premature and largely based on limited evidence. Most existing data on host restriction stem from fish alloherpesviruses [40].

Only a single *Bd*-positive individual was detected, and no ranaviruses were found. However, the small sample size for species other than *B. bufo* limits broader interpretation. The low *Bd* detection rate in our toad samples is consistent with known host-specific infection patterns, as *B. bufo* tend to show low susceptibility to *Bd* [23]. Data from concurrent live animal sampling in the same region indicate higher *Bd* prevalence and support the absence of ranaviruses in northern Germany (Striewe et al., unpublished data). These findings suggest that carcass-based surveillance may underestimate the true infection rates of some pathogens, particularly those with low host mortality or low detectability in roadkill specimens.

The samples used in this study were collected over an extended period of time. Storage duration ranged greatly and reached up to 400 days. However, our data do not indicate storage-related bias in PCR detection. Moreover, successful detection of RaHV3 in two samples stored for up to 399 days aligns with previous studies demonstrating long-term stability of DNA for PCR-based analyses [41,42].

Most specimens in this study were road-killed animals collected during breeding migrations, resulting in a strong bias toward *B. bufo*. This sampling strategy precludes reliable inference on population-level prevalence and limits conclusions on pathogen detection within the submitted carcass material. This sampling bias likely explains apparent associations between virus detection and species, sex, or roadkill status, which disappeared when controlling for sample size. Although pathogen detection was significantly associated with species in the full dataset, this effect was driven by the dominance of *B. bufo* in the sample and the absence of positive cases in other species. Therefore, no conclusions regarding species-specific susceptibility can be drawn from the present dataset. Male *B. bufo* are known to be disproportionately affected by road mortality during breeding migrations [43], and sex-related patterns in carcass-based datasets may therefore reflect collection bias rather than true sex-specific susceptibility. As carcasses from wetlands or forested habitats are seldom recovered due to scavenging and inaccessibility [24], roadkill remains a practical but biased sample source.

Passive surveillance based on opportunistically collected carcasses can provide preliminary insights into pathogen presence and geographic distribution, particularly through molecular detection methods. However, such data are inherently limited by sample degradation, contamination risk, and strong sampling bias, and therefore cannot replace active surveillance or support detailed pathological or population-level inferences. Instead, carcass-based surveillance should be regarded as a complementary, non-invasive screening approach to identify pathogen occurrence and inform targeted follow-up investigations.

## Figures and Tables

**Table 1:** Detection of *Batrachochytrium dendrobatidis* (*Bd*), bufonid herpesvirus 1 (BfHV1), and ranid herpesvirus 3 (RaHV3) in *Bufo bufo* (common toads) and *Rana temporaria* (common frogs) from Schleswig-Holstein, showing sample size (*n*), number of positives (positive), and percentage of positives with 95% confidence intervals (% positive (95% CI).

Species	*Bd*			BfHV1			RaHV3		
	*n*	Positive	% Positive (95% CI)	*n*	Positive	% Positive (95% CI)	*n*	Positive	% Positive (95% CI)
*B. bufo*	148	1	0.7 (0.0–3.7)	148	72	48.6 (40.4–57.0)	148	1	0.7 (0.0–3.7)
*R. temporaria*	19	0	0.0 (0.0–17.6)	19	0	0.0 (0.0–17.6)	19	5	26.3 (9.1–51.2)

**Table 2:** Detection of bufonid herpesvirus 1 (BfHV1) DNA in skin, kidney, liver, and brain tissues from *Bufo bufo* (common toads), showing the number tested (*n* tested), number positive (*n* positive), proportion of positive results, corresponding 95% confidence intervals (CIs), and the conditional proportion among individuals testing positive in at least one tissue.

Tissue	*n* Tested	*n* Positive	% Positive	95% CI	Conditional Proportion (%)
Skin	147	71	48.3	40.4–57.0	98.6
Kidney	49	13	26.5	14.9–41.8	54.2
Liver	47	9	19.1	9.2–33.3	37.5
Brain	25	6	24	9.4–45.1	42.9

**Table 3:** Analysis of associations between pathogen detection (*Batrachochytrium dendrobatidis* (*Bd*), bufonid herpesvirus 1 (BfHV1), and ranid herpesvirus 3 (RaHV3) and selected variables in *Bufo bufo* (common toads) only. Factors included carcass origin, decomposition state, storage duration (days) and sex. Test type, raw *p*-values, and Benjamini–Hochberg–adjusted *p*-values (FDR) are reported.

Pathogen	Factor	Test Type	*p*-Value	FDR
BfHV1	Roadkill	Fisher	0.404	0.799
	Decomposition state	Chi-square	0.743	0.799
	Storage duration	Wilcoxon	0.201	0.302
	Sex	Chi-square	0.565	0.799
RaHV3	Roadkill	Fisher	0.097	0.788
	Decomposition state	Chi-square	0.794	0.799
	Storage duration	Wilcoxon	0.143	0.303
	Sex	Chi-square	0.377	0.799
*Bd*	Decomposition state	Chi-square	0.794	0.799
	Storage duration	Wilcoxon	0.201	0.302
	Sex	Chi-square	0.377	0.799

## Data Availability

The original contributions presented in the study are included in the article; further inquiries can be directed by the corresponding author.
